# Global marine protected areas do not secure the evolutionary history of tropical corals and fishes

**DOI:** 10.1038/ncomms10359

**Published:** 2016-01-12

**Authors:** D. Mouillot, V. Parravicini, D. R. Bellwood, F. Leprieur, D. Huang, P. F. Cowman, C. Albouy, T. P. Hughes, W. Thuiller, F. Guilhaumon

**Affiliations:** 1 UMR 9190 MARBEC, IRD-CNRS-IFREMER-UM, Université de Montpellier, Montpellier 34095, France; 2 Australian Research Council Centre of Excellence for Coral Reef Studies, James Cook University, Townsville, Queensland 4811, Australia; 3 CRIOBE, USR 3278 CNRS-EPHE-UPVD, Labex ‘Corail', University of Perpignan, Perpignan 66860, France; 4 Department of Biological Sciences and Tropical Marine Science Institute, National University of Singapore, Singapore 117543, Singapore; 5 Department of Ecology & Evolutionary Biology, Yale University, 21 Sachem St, New Haven, Connecticut 06511 USA; 6 Département de biologie, chimie et géographie, Université du Québec à Rimouski, 300 Allée des Ursulines, Rimouski, Canada G5L 3A1; 7 Laboratoire d'Écologie Alpine (LECA), Univ. Grenoble Alpes, Grenoble F-38000, France; 8 Laboratoire d'Écologie Alpine (LECA), CNRS, Grenoble F-38000, France

## Abstract

Although coral reefs support the largest concentrations of marine biodiversity worldwide, the extent to which the global system of marine-protected areas (MPAs) represents individual species and the breadth of evolutionary history across the Tree of Life has never been quantified. Here we show that only 5.7% of scleractinian coral species and 21.7% of labrid fish species reach the minimum protection target of 10% of their geographic ranges within MPAs. We also estimate that the current global MPA system secures only 1.7% of the Tree of Life for corals, and 17.6% for fishes. Regionally, the Atlantic and Eastern Pacific show the greatest deficit of protection for corals while for fishes this deficit is located primarily in the Western Indian Ocean and in the Central Pacific. Our results call for a global coordinated expansion of current conservation efforts to fully secure the Tree of Life on coral reefs.

## Abstract (web summary)

Marine protected areas (MPAs) are established to conserve species, but the extent to which they also conserve evolutionary history is not clear. Here, Mouillot *et al*. show that for tropical corals and fish, the current global MPA network secures only 1.7 and 17.6% of phylogenetic diversity, respectively.

## Graphical abstract

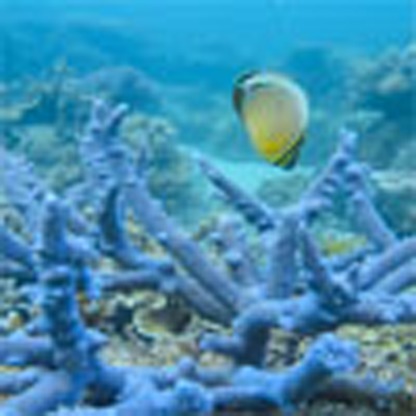

Human activities are altering ecosystems worldwide, changing their biodiversity and composition, and imperilling their capacity to deliver ecosystem services^1^. In this context, protected areas are indisputably the flagship tool for protecting both ecosystems and biodiversity by limiting direct human impacts^2^. Conservation strategies have traditionally focused on vulnerable components of taxonomic diversity such as endemic, rare or threatened species^3^,^4^. However, phylogenetic diversity, represented by the Tree of Life, is becoming an increasingly important component of conservation science^5^,^6^ since it represents the breadth of evolutionary history^7^ and supports biodiversity benefits and uses, often unanticipated, for future generations^8^,^9^. Phylogenetically related species tend to have similar functional traits, environmental niches and ecological interactions^10^,^11^, although numerous counter examples exist^12^,^13^. Therefore, species that are more phylogenetically distinct may have greater functional complementarity. In turn, species assemblages that are more phylogenetically diverse may promote greater biomass production within^14^ and across^15^ trophic levels even though a universal relationship between phylogenetic diversity and ecosystem functioning remains questionable^9^,^16^. Yet, few studies have quantitatively assessed the extent to which protected areas encompass phylogenetic diversity^17^,^18^ and none have focused on marine taxa at a global scale.

Here, we tackle this critical issue for the iconic but threatened coral reefs of the world that support one of the largest concentrations of biodiversity, around 830,000 multi-cellular species^19^, and provide vital ecosystem services to half a billion people including food security^20^, financial incomes^21^ and protection against natural hazards^22^. There is overwhelming evidence that human activities, particularly fishing pressure and pollution, affect coral reef ecosystem state^23^, functioning^24^ and resilience^25^. Thus, to counteract human impacts and maintain the integrity of coral reefs, thousands of marine-protected areas (MPAs) have been created worldwide^26^. However, the spatial design of the global MPA system is largely contingent on local socioeconomic conditions and history rather than regional or global considerations^27^,^28^. Furthermore, given the limited resources dedicated to conservation efforts^29^ and the need to maintain coastal fisheries for people's livelihoods^21^,^30^, MPAs cannot be extended to all coral reefs. Guiding future conservation strategies thus remains a key challenge, particularly at a global scale where deficits of protection must be identified and addressed to achieve effective protection of evolutionary history on coral reefs. Here we assessed the extent to which the global system of MPAs represents individual species and phylogenetic diversity for two major components of coral reef ecosystems, shallow-water corals in the order Scleractinia (805 species) and fishes in the family of Labridae (452 species). These groups contribute to the high biodiversity of tropical seas^31^ and help maintaining productive and resilient reefs^32^,^33^. We show that the current global MPA system, covering 5.9% of the world's coral reef area, does not meet the minimum conservation targets considered necessary to adequately secure the branches of the Tree of Life for corals or fishes, particularly the longest branches that represent the greatest amount of evolutionary history.

## Results and Discussion

### Lag behind minimum conservation targets

**Figure 1:**
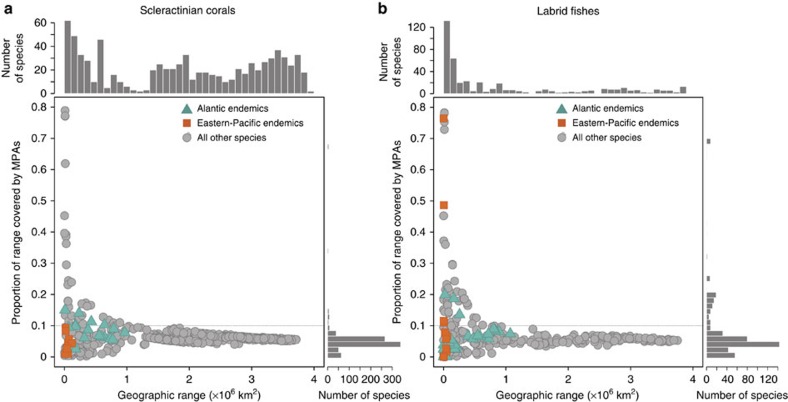
Relationship between the total geographic range of species and the proportion of that range covered by the global system of MPAs. (**a**) Scleractinian coral species and (**b**) fish species of the family Labridae. Histograms on top and to the right represent the distributions of total ranges and proportion of protection among species respectively. Coloured squares and triangles represent endemic species, that is, only present in one of the two biogeographic realms: Atlantic and Eastern Pacific, respectively. Dotted lines represent the 10% threshold corresponding to the minimum representation target for sustaining species persistence.

Using global distribution maps of each scleractinian coral and labrid fish species (Methods), we reveal that only 5.7% of coral species and 21.7% of fish species meet a minimum protection target of 10% potential coverage of their geographic range by the global system of MPAs (Fig. 1). Regionally, the situation is even more contrasted. For example, coral species that occur exclusively in the Tropical Eastern Pacific all fall below the critical 10% coverage threshold. Similarly, all coral and fish species found only in the Atlantic have <20% coverage (Fig. 1). This 10% threshold has been specifically advocated for wide-ranging species (>250,000 km^2^) and is regarded as a conservative target of coverage by protected areas for sustaining species persistence^3^,^34^. This conservative cut-off takes into account commission errors, that is, the potential absence of a given species from protected areas that lie within its geographic distribution due to chance or unsuitable habitats^34^.

**Figure 2:**
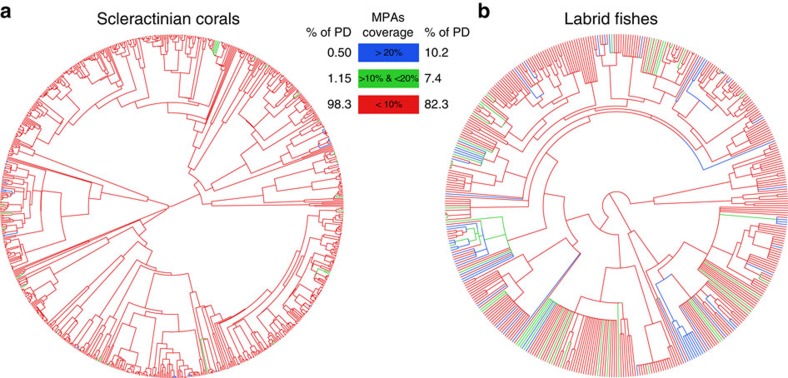
Percentages of geographic ranges covered by the global system of MPAs for species and internal branches across the Tree of Life. (**a**) Scleractinian coral species and (**b**) fish species of the family Labridae. Species or branches in red do not meet the minimum 10% representation threshold, that is, <10% of their geographic range is covered by MPAs, while green and blue colours indicate 10–20% and more than 20% coverage respectively. The corresponding percentage of total phylogenetic diversity (PD) is indicated for each coverage category.

By applying the same reasoning to the internal branches of the phylogenetic trees (Methods), we show that only 1.7% (±0.2 s.d.) of the Tree of Life of corals and 17.6% (±0.6 s.d.) of fishes attain the minimum 10% coverage (Fig. 2). Thus 7,160 Myr of the evolutionary history of corals and 3,586 Myr of fishes are inadequately represented by the global MPA system, far more than for many other threatened taxonomic groups^8^. Globally, the amount of evolutionary history potentially covered by MPAs, that is, the proportion of the geographic range of evolutionary branches overlapping with the global MPA system, is only 6.0% (±0.1 s.d.) and 8.7% (±0.2 s.d.) for corals and fishes, respectively. Coral evolutionary history receives significantly less coverage than expected under a random distribution of species geographic ranges across the Tree of Life (*P*<0.001, *n*=999, randomization test) while fishes receive significantly more protection than expected by chance (*P*<0.001, *n*=999, randomization test) (Methods). The greatest amount of evolutionary history is supported by the longest branches on the Tree of Life. In our case, the top 10% longest extant and internal branches, corresponding to >8.68 Myr (±0.5 s.d.) for corals and >10.7 Myr (±0.25 s.d.) for fishes, support a disproportional amount of evolutionary history, with 62% (±0.9% s.d.) and 34% (±0.5% s.d.) for corals and fishes, respectively. These longest branches are overwhelmingly under-represented within the global MPA system (Fig. 2). Only 1.3% (±0.6% s.d.) of the longest branches in corals and 20.2% (±2.3% s.d.) in fishes are adequately protected by the minimum threshold of 10% geographic coverage by MPAs. If those poorly protected longest branches support endangered species we may expect large and abrupt changes in ecosystem functioning following extinctions. This situation already exists for the world's primates, where the most endangered species are both evolutionarily and ecologically distinct^35^. In the sea global extinctions remain scarce, partly due to limited assessment^36^, but the functionally most distinctive fish species on coral reefs tend to be rare either in their geographic extent or their local abundance^37^. We may thus anticipate a disproportional local loss of functional diversity within coral reef communities if the longest evolutionary branches are under threat and inadequately protected^38^. For instance long-branched lineages include relatively specialized forms, such as the large invertivore *Lachnolaimus* and the world's largest excavating parrotfsh *Bolbometopon* which are severely overexploited, suggesting that the loss of long branches may result in the loss of unique and functionally important groups^39^.

### Global distribution of protection deficits

**Figure 3:**
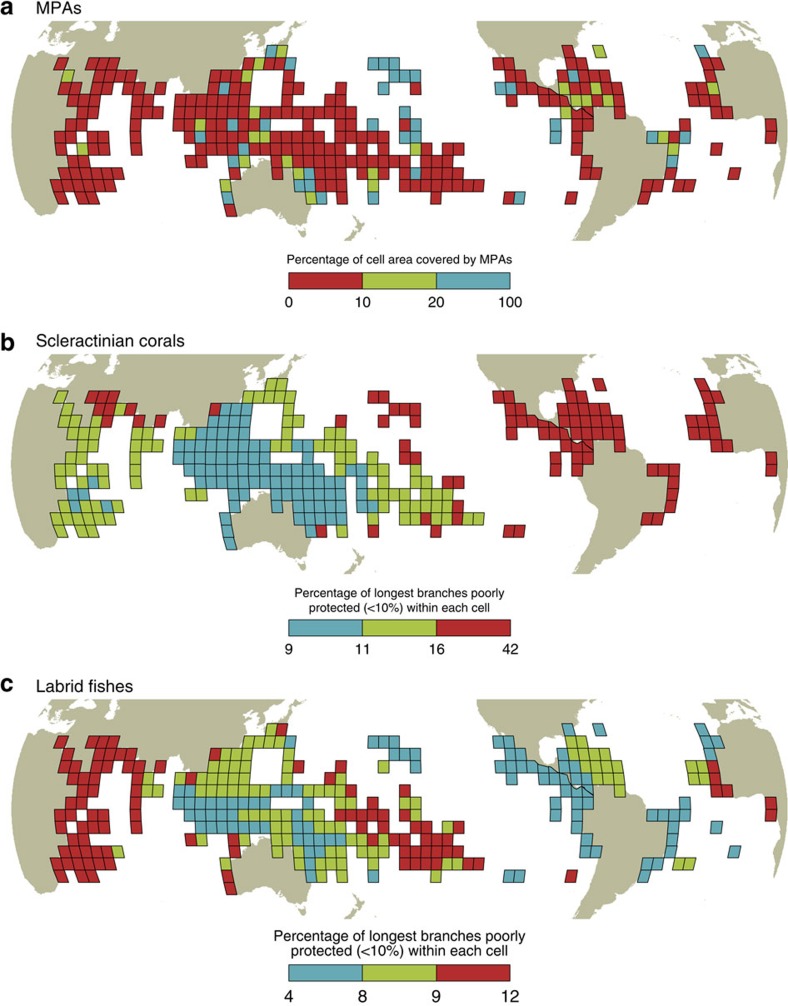
Global distribution of protection deficits to secure the Tree of Life on coral reefs. Global maps representing, for each cell (5° × 5°), the percentage of coral reef habitat covered by MPAs (**a**), and the proportion of the longest evolutionary branches (top 10%) that receive less than the critical 10% coverage by the MPA system within coral (**b**) and fish (**c**) local assemblages. Colours correspond to three categories of values based on percentage of coverage for MPAs and on tertiles for corals and fishes.

**Figure 4:**
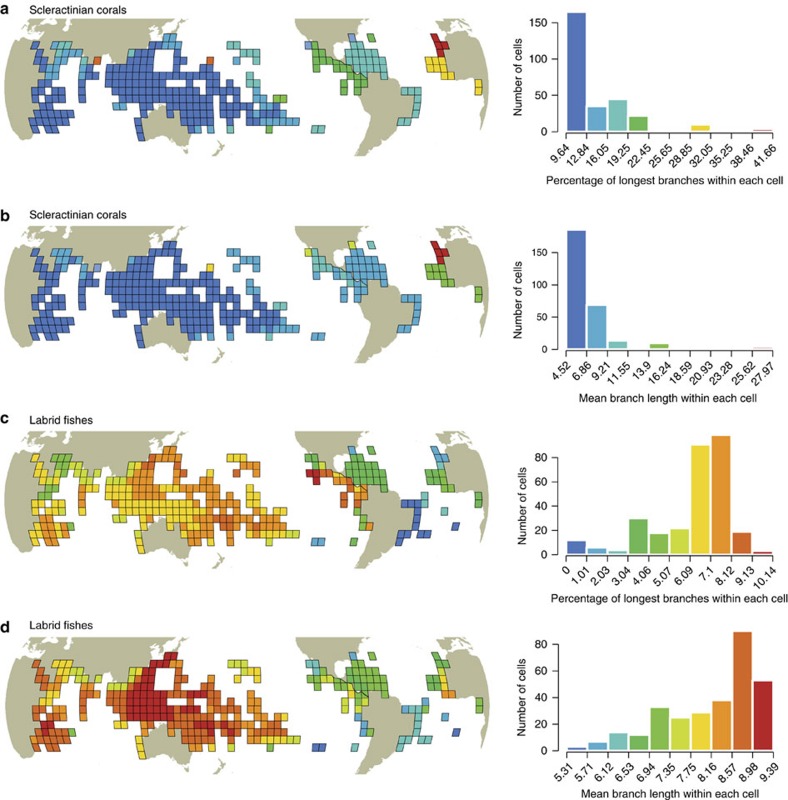
Global distribution of the amount of evolutionary history on coral reefs. Global maps representing, for each grid cell (5° × 5°), the percentage of the longest evolutionary branches (top 10%) and the mean evolutionary branch length within coral (**a**,**b**) and fish (**c**,**d**) local assemblages, respectively. Colours correspond to classes of the histograms representing the distribution of values across the cells.

**Figure 5:**
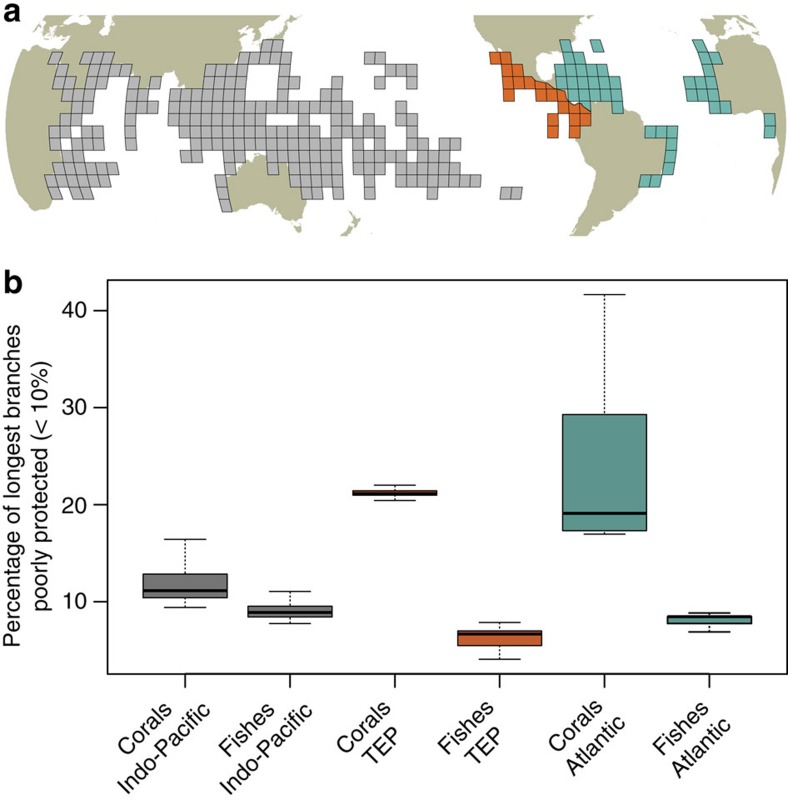
Representation in MPAs for branches of the Tree of Life on coral reefs across marine realms. (**a**) Global map representing the three marine realms: Indo-Pacific (grey), Tropical Eastern Pacific (orange), and Atlantic (green). (**b**) Boxplots (median and quartiles) representing the percentage of the longest evolutionary branches (top 10%) that receive less than the critical 10% coverage by the MPA system within coral and fish local assemblages (in 5° × 5° grid cells) of the three marine realms.

To highlight the critical gaps in protecting the Tree of Life on coral reefs, we mapped the locations where the longest evolutionary branches that receive <10% coverage are concentrated using a regular grid of 5° × 5° cells (Methods). For corals, the longest evolutionary branches with low protection are predominantly in the Atlantic, Eastern Pacific and, to a lesser extent, the North Indian Ocean (Fig. 3b). These deficits of protection are only marginally correlated with the heterogeneous MPA coverage at the global scale (*r*=0.045, *n*=304 5° × 5° grid cells, *P*>0.05, Fig. 3a). Instead, the high proportion of longest branches, and their unique evolutionary history, in the Atlantic and Eastern Pacific primarily drives this pattern^40^ (Fig. 4a,b). For fishes, the highest concentrations of poorly protected long branches are located in the Western Indian, Central Pacific and, to a lesser extent, the Eastern Atlantic (Fig. 3c). As in corals, these deficits of protection are not correlated with the heterogeneous distribution of MPA coverage (*r*=0.025, *n*=287 grid cells, *P*>0.05, Fig. 3a). Instead, the pattern is driven by the relatively high proportion of long evolutionary branches of fishes at the periphery of the Indo-Pacific^41^ (Fig. 4c,d). The correlation between the proportion of poorly protected longest evolutionary branches for corals and fishes within assemblages is negative (*r*=−0.15; *n*=287 grid cells, *P*=0.30) suggesting that there is a global spatial mismatch, albeit weak, of conservation needs for these two taxa. The Atlantic and Eastern Pacific tend to concentrate many long and poorly protected branches for corals but substantially less for fishes (Fig. 5). This most likely reflects the biogeographic history of the tropical Atlantic which has been characterized by isolation, thus maintaining old coral lineages^40^ in contrast to the recent diversification in younger fish lineages^42^, especially along the Brazilian coast where there is extensive evidence of recent colonization^43^. In the Atlantic, therefore, there is a logical priority to emphasize the protection of older coral lineages. For fishes, the Atlantic hosts younger labrid lineages than the Indo-Pacific particularly in the Caribbean following cryptic speciation^42^ and in the North Eastern Atlantic with subsequent diversification of Mediterranean lineages following the Messinian Salinity Crisis at 6 Myr (ref. 44). By contrast, the Coral Triangle, at the centre of the Indo-Pacific region, harboured most of the coral reef refugia during the Quaternary glaciations, hence acting as a ‘museum' for the older labrid lineages^45^.

Globally, the proportion of poorly protected longest branches in corals ranges from 9 to 42% compared with 4 to 12% in fishes (Fig. 3b,c), suggesting that conservation efforts should initially be focused on the Atlantic to better preserve the coral Tree of Life where it is most at risk. West African and, to a lesser extent, South American countries that border each side of the Atlantic, show the slowest rate of MPA establishment worldwide although positive outliers in environmental governance also occur at both national and local levels^28^. For example, the Dominican Republic has already reached the target of 10% coverage. Similarly an increase in conservation investment has promoted MPA establishment in Eastern Africa^46^. Other countries of Western Africa and Eastern America remain far below the 10% coverage and should be priority areas to better protect the evolutionary history of corals. For fishes, conservation investment are primarily needed in the Western Indian Ocean where poorly protected longest branches are concentrated.

### Limitations and less conservative protection assessment

Overall, our results show that the Tree of Life on coral reefs is inadequately represented by the current global MPA system, with most evolutionary branches, particularly the longest ones, receiving <10% protection. Despite the magnitude of this shortfall, our estimates are highly conservative because they are based on the assumption that all MPAs are able to protect every coral and fish species that geographically overlaps with them. It thus assumes that coral and fish species are present in all MPAs within their geographic ranges, and that all MPAs are effective in their protection. These assumptions may not be valid. First, we have no proof of individual species presence within MPAs. These commission errors are inevitable given the coarse grain of species geographic distributions and the small size of most MPAs. We therefore assess maximum potential protection while the conservation target of 10% is partly set to compensate for this limitation^34^. Second, although there is overwhelming evidence that MPAs can maintain or increase fish diversity, size and biomass^47^,^48^, and strong evidence that the presence of intact fish communities can enhance coral persistence and recovery^49^,^50^,^51^, the extent of these benefits may vary among MPAs. Not all MPAs are able to ensure that fish and coral communities are protected, due to poor compliance and enforcement^52^. Furthermore, MPAs cannot prevent pulses of coral mortality from cyclones or coral bleaching^53^, or from chronic declines in coral recruitment and growth due to degraded water quality^54^,^55^. MPAs in the Atlantic should better focus on coral lineages while those in the Western Indian Ocean should primarily limit fish overexploitation to protect the amount of evolutionary history on coral reefs. If we exclude MPAs that are not specifically designed to protect species and habitats and have a reduced capacity to protect fish diversity and biomass^48^, that is, if only IUCN categories I to IV are considered (Methods), the proportion of the Tree of Life attaining the minimum target of 10% coverage by MPAs drops to 0.9% (±0.2 s.d.) and 14.9% (±2.0 s.d.) for corals and fishes, respectively.

## Conclusions

Phylogenetic diversity is one of the key components of biodiversity^5^,^14^. However, the existing global system of MPAs does not meet the minimum levels considered necessary to adequately protect the Tree of Life for corals or fishes. If MPAs are to protect the Tree of Life, we need to carefully consider their features and future placement. Geographic variation in evolutionary history, and variable susceptibility to human impacts differs among fish and corals. The most notable example is in the Atlantic where there is a predominance of old coral lineages but a larger proportion of younger fish lineages. This mismatch brings to the fore the potential limitations of MPAs, and the differing needs of fishes, corals and other taxa. For corals, many of the major ongoing threats are not mitigated by MPAs. For effective protection we may need to look beyond traditional MPAs and develop new strategies that can encompass the full range of threats to reef biodiversity. A broader approach could include the protection of herbivorous fishes that promote local recovery of corals^50^, management to control terrestrial influences and water quality^56^ and effective action to mitigate climate change^57^. For future conservation efforts, we need to adequately secure greater amounts of evolutionary history on coral reefs in the Atlantic, Eastern Pacific and in the Western Indian Ocean.

## Methods

### Data

We restricted our database to shallow reef habitats (<50 m) showing a minimum monthly sea surface temperature (hereafter SST) of at least 17 °C to define tropical marine waters^58^. We built the geographic distribution of 452 tropical reef fish species from the Family Labridae by compiling 455 references from 169 locations worldwide^58^. From these distributional data we obtained a range map for each species, defined as the convex polygon shaping the area where each species is present^58^. These were individually checked by expert to avoid the combination of disjointed ranges, for example, anti-tropical species.

We focused on labrid fishes since they (i) represent an exceptionally rich and diverse reef associated family, (ii) live in shallow waters, (iii) benefit from MPAs^59^ as a common fisheries target^24^ and (iv) have a well resolved phylogeny^41^. To incorporate unsampled taxa, new tips were grafted onto a backbone phylogeny based on other published phylogenies for the group^60^,^61^, supplemented by species accounts from fish identification guides and FishBase (www.fishbase.org). Where information allowed, new tips representing unsampled species were added to direct sister species or to the base of the clade representing its genus. The full list of labrid fishes is provided as Supplementary Data 1.

We selected 805 coral species for which global range maps were downloaded at http://www.iucnredlist.org/technical-documents/spatial-data#corals. We considered only hard corals in shallow habitats. We used the supertree method to reconstruct the phylogeny of the scleractinian clade, comprising a total of 842 reef and 705 non-reef species^40^. The source trees were derived from a molecular phylogeny of 474 species (based on seven mitochondrial DNA markers), 13 morphological trees and 1 taxonomic tree. These were combined via the SuperFine-boosted Matrix Representation with Parsimony^62^ and Matrix Representation with Likelihood^63^. The full list of coral species is provided as Supplementary Data 2.

We collected spatial information on MPAs from the WDPA (World Database on Protected Areas) database available at: http://protectedplanet.net/. The original database included 9,600 PAs covering a total surface of 17,633,881 km^2^. We eliminated PAs on land, those that did not involve coastal habitat, defined as the portion of sea bottom from 0 to 200 m depth, and MPAs designated to protect species not considered in the present study (for example, birds). The latter were discarded after evaluating the description of the ‘Designation' field in the original IUCN-WDPA database. MPAs for which IUCN criteria were either ‘not applicable' or ‘unknown' (for example, not communicated by the Authority), and are likely to be unreliable, were also removed. The final database included 3,625 MPAs covering a total surface of 942,568 km^2^ (IUCN categories I–VI). We also used another restricted data set where we eliminated MPAs that are not specifically designed to protect species or habitats. We retained the 2,224 MPAs belonging to IUCN categories I to IV covering a total surface of 575,806 km^2^ with a relatively higher degree of protection.

We then used a 5° × 5° grid cell corresponding to ∼550 × 550 km at the equator to collate the presence of species, the area of tropical reef habitat, and the area of reef habitat protected within MPAs^64^.

### Analyses

Fossil records show that species extinction risk is primarily determined by geographic range size in the marine realm^65^,^66^ with restricted ranged species being less buffered against demographic variability under changing environments. However, having at least ‘one foot' in the MPA system does not ensure persistence^67^. We thus examined the proportion of the geographic range of species overlapping with the global MPA system. This represents a potential overlap since the presence of species within MPAs overlapping with their geographic range was not measured directly. We adopted a threshold of 10% spatial coverage by MPAs corresponding to a minimum (and conservative) target for effective protection^3^,^34^. This minimum threshold is based on the rational that some MPAs may be unsuitable for a given species, that protection is not effective in all MPAs and that the coarse grain of species distribution maps may induce commission errors by which species can be absent from protected areas that overlap their geographical ranges^34^.

We applied the same reasoning to the internal branches of phylogenetic trees. The coverage by MPAs of the evolutionary history of a branch is therefore defined as the relative coverage by MPAs of the combined geographic ranges of the species subtending this branch. To evaluate the effectiveness with which MPAs protect the overall Tree of Life we measured the amount of evolutionary history represented by branches that pass the coverage threshold of 10%.

By grafting species we create polytomies on the phylogenetic trees that may bias the results since many species have artificially identical branch lengths. This may ultimately inflate the amount of evolutionary history supported by the tips and the level of phylogenetic conservatism^68^. To limit this bias and estimate the uncertainty of our results linked to the unresolved recent diversification events, polytomies were randomly resolved by a birth–death model^69^ using BEAST^70^. Using 100 resolved trees for both corals and fishes, we provided the mean value and s.d. (±) for each result.

We also tested whether the current global system of MPAs is effective given the topology of the phylogenetic tree and thus the evolutionary constraints that have shaped species geographic ranges across history. To do so we performed a null model analysis where species labels were shuffled across the tips of the two phylogenies. By so doing the null model breaks the relationships between species ranges and their position on the phylogenetic tree while maintaining the amount of species coverage by MPAs. This procedure was applied 999 times for each of the 100 resolved trees to obtain a null frequency distribution for the overall amount of evolutionary history covered by MPAs. From this distribution, we extracted a *P* value for each resolved tree by assessing the positions of the observed in the null frequency distribution. These 100 *P* values were combined using the Fisher's combined probability test to provide a global *P* value quantifying whether the current global system of MPAs is more or less effective for the observed distribution of species geographic ranges across the phylogenies when compared to a random distribution.

To highlight the critical geographical gaps in protecting the Tree of Life on coral reefs, we mapped, at the grid cell level, the proportion of the longest evolutionary branches that receive <10% coverage. The longest branches are the top 10% for each Tree of Life. We also mapped the proportion of the longest evolutionary branches that are poorly protected, and the mean length of evolutionary history in each grid cell, that is, at the species assemblage level, and in each realm for both corals and fishes.

## Additional information

**How to cite this article:** Mouillot, D. *et al*. Global marine protected areas do not secure the evolutionary history of tropical corals and fishes. *Nat. Commun.* 7:10359 doi: 10.1038/ncomms10359 (2016).

## Supplementary Material

Supplementary Data 1 Fish species list with their characteristics.

Supplementary Data 2 Coral species list with their characteristics.
